# Does ^18^F-FDG PET/CT add value to conventional imaging in clinical assessment of chronic disseminated candidiasis?

**DOI:** 10.3389/fmed.2022.1026067

**Published:** 2022-12-20

**Authors:** Blandine Rammaert, Christophe Maunoury, Tioka Rabeony, Jean-Michel Correas, Caroline Elie, Serge Alfandari, Pierre Berger, Marie-Thérèse Rubio, Thorsten Braun, Prissile Bakouboula, Sophie Candon, Françoise Montravers, Olivier Lortholary

**Affiliations:** ^1^Université de Paris Cité, APHP, Service des Maladies Infectieuses et Tropicales, Hôpital Necker-Enfants Malades, Centre d’Infectiologie Necker-Pasteur, Institut Imagine, Paris, France; ^2^Université de Paris, APHP, Hôpital Européen Georges Pompidou, Service de Médecine Nucléaire, Paris, France; ^3^APHP, URC Necker-Cochin, Paris, France; ^4^Université de Paris Cité, APHP, Service de Radiologie Adulte, Hôpital Necker-Enfants Malades, Paris, France; ^5^Centre Hospitalier Tourcoing, Service de Réanimation et Maladies Infectieuses, Tourcoing, France; ^6^Institut Paoli-Calmettes, Infectiologie Transversale, Marseille, France; ^7^CHU Nancy, Service d’Hématologie, Nancy, France; ^8^Université de Paris Nord, APHP, Hôpital Avicenne, Service d’Hématologie, Bobigny, France; ^9^Université de Rouen Normandie, INSERM U1234, CHU de Rouen Normandie, Rouen, France; ^10^Sorbonne Université, APHP, Service de Médecine Nucléaire, Hôpital Tenon, Paris, France; ^11^Institut Pasteur, CNRS, Unité de Mycologie Moléculaire, Centre National de Référence Mycoses Invasives et Antifongiques, UMR 2000, Paris, France

**Keywords:** invasive fungal disease, Candida, candidiasis, hematological malignancy, immune reconstitution inflammatory syndrome, acute leukemia, hematopoietic stem cell transplantation

## Abstract

**Background:**

Chronic disseminated candidiasis (CDC) classically occurs after profound and prolonged neutropenia. The aim of the CANHPARI study was to assess the clinical value of adding ^18^F-fluorodeoxyglucose PET/CT to conventional radiology for initial and subsequent evaluations of CDC.

**Materials and methods:**

A pilot prospective study was conducted in 23 French onco-hematological centers from 2013 to 2017 (NCT01916057). Patients ≥ 18 y.o. suspected for CDC on abdominal conventional imaging (CT or MRI) were included. PET/CT and conventional imaging were performed at baseline and month 3 (M3). Follow-up was assessed until M12. The primary outcome measure was the global response at M3, i.e., apyrexia and complete response to PET/CT. The secondary outcome measure consists in comparison between responses to PET/CT and conventional imaging at diagnosis and M3.

**Results:**

Among 52 included patients, 44 were evaluable (20 probable and 24 possible CDC); 86% had acute leukemia, 55% were male (median age 47 years). At diagnosis, 34% had fever and conventional imaging was always abnormal with microabscesses on liver and spleen in 66%, liver in 25%, spleen in 9%. Baseline PET/CT showed metabolic uptake on liver and/or spleen in 84% but did not match with lesion localizations on conventional imaging in 32%. M3 PET/CT showed no metabolic uptake in 13 (34%) patients, 11 still having pathological conventional imaging. Global response at M3 was observed in eight patients.

**Conclusion:**

Baseline PET/CT does not replace conventional imaging for initial staging of CDC lesions but should be performed after 3 months of antifungal therapy.

**Clinical trial registration:**

[www.clinicaltrials.gov], identifier [NCT01916057].

## Introduction

Chronic disseminated candidiasis (CDC), often referred to as hepatosplenic candidiasis, is a rare disease occurring mostly in hematological malignancies with an incidence rate estimated at 1.53 per 100 patient-years (1, 2). Early and appropriate diagnosis and treatment of CDC are crucial, since anti-tumor chemotherapy or hematopoietic stem cell transplantation (HSCT) delay can negatively affect the underlying prognosis (3). Current IDSA Candida guidelines recommend for CDC a lipid formulation of amphotericin B or an echinocandin for several weeks followed by oral fluconazole in the case of azole-susceptible *Candida* isolate. Therapy should be protracted until lesions resolve on repeated imaging (4). It should at least last 8 weeks according to ESCMID guidelines (5). However, in a retrospective Taiwanese study 45.7% of CDC patients had residual liver and/or spleen abscesses on imaging at 6 months (2). Hence, prolonged treatments up to 3–6 months are frequent, leading to antifungal toxicity and cost increase (2, 6, 7). Finally, our group advocated that CDC belonged to the spectrum of fungal immune reconstitution inflammatory syndrome (IRIS), thereby raising questions on the tools most appropriate to evaluate the CDC-induced inflammatory reaction over time (8–10).

F18-fluorodeoxyglucose positron-emission tomography computed tomography (PET/CT) has shown a positive impact on reducing duration of antimicrobial therapy during febrile neutropenia in hematological patients (11). Lesions due to various fungi such as *Aspergillus* spp., *Mucorales*, *Cryptococcus* spp., *Histoplasma* spp., *Coccidioides* spp. are able to metabolize F18-fluorodeoxyglucose (12–15). Indeed, a preliminary study by our team has assessed PET/CT as a diagnostic tool for invasive fungal diseases (IFD), including CDC (16), its appropriateness being further documented by descriptive case reports or small case series uptake (17–21). PET/CT might therefore help to assess lesions at baseline and monitor antifungal treatment duration of CDC (9, 10). Through the largest prospective study on CDC ever performed (10), the aim was to determine whether the addition of PET/CT to conventional radiology added clinical value both at diagnosis and after 3 months of antifungal therapy.

## Patients and materials and methods

### Study design and population

CANHPARI was a multicenter pilot prospective interventional study designed to determine whether PET/CT is useful for treatment strategy for CDC. The study was registered on clinicaltrials.gov (NCT01916057) and approved by the ethic committee (CPP Ile de France 1, 2013-mai-13239).

Eligible patients met the following criteria: aged ≥ 18 years with a malignancy or HSCT, and profound, i.e., < 100 neutrophils/mm^3^, and prolonged, i.e., ≥ 10 days, neutropenia; and CDC defined by multiple abscesses in liver and/or spleen observed on abdominal CT or MRI (22). Exclusion criteria were described elsewhere (22). After file assessment by an independent committee, consisting in two infectious disease specialists, two hematologists, and two nuclear medicine specialists, patients were excluded if they had liver and/or lesions from an origin other than candidiasis. Since the definition of IFD changed long after the beginning of the study (23), CDC were categorized as proven, probable, or possible according to the previous 2008 EORTC/MSG criteria (22).

After providing written informed consent, all recruited patients benefited from PET/CT according to the European Association of Nuclear medicine guidelines within a week following inclusion and after 3 months of antifungal therapy (M3) (24). If PET/CT was performed for diagnosis before inclusion, no subsequent PET/CT was performed at baseline. Conventional imaging (CT or MRI) as part as routine follow-up was performed at diagnosis, M3, and M6. The follow-up was scheduled for 12 months.

Computed tomography (CT)-guided or ultrasonography-guided liver biopsy was performed at the physician’s discretion. Beta-1,3-D-glucans (BDG; Fungitell^®^, Beacon Diagnostics, East Falmouth, MA, USA) were considered present when one result was ≥ 80 pg/mL. Antifungal prophylaxis was considered if taken for at least 5 days, time corresponding of study state of most antifungal drugs.

### Primary outcome measure

Global response at M3 was defined by a combined criterion, resolution of fever attributable to CDC and complete response to PET/CT. Criteria defining responses to therapy and study outcomes have been validated in interventional studies including therapeutic trials, therefore not applicable to the present study (10, 25). Consequently, clinical, biological and radiological criteria were adapted leading to the response definitions presented in Table 1. SUVs are not relevant in this study since PET/CT have been performed on different devices from different manufacturers, according to the high number of involved centers. To avoid this bias, we chose to perform a visual analysis to describe baseline PET/CT: any focal uptake above the uninvolved liver or splenic background activity was considered as a candidiasis lesion. Since there was no gold standard for PET/CT criteria to assess infectious complications, a complete response to PET/CT was defined by the visual disappearance of all target hypermetabolic lesions, according to PET Response Criteria in Solid Tumors (PERCIST) (26) (for more details see Supplementary Table 1). Global response at M3 was assessed by the independent committee. Each PET/CT was independently reviewed and analyzed by two experienced nuclear medicine physicians blinded to the patient’s history and any discrepancy was further resolved by consensus.

**TABLE 1:** Adapted criteria for chronic disseminated candidiasis (CDC) outcome.

Response	Type of response	Criteria
Clinical response	Complete	Survival and resolution of all attributable symptoms and signs (i.e., fever, digestive symptoms, hepatomegaly, splenomegaly) of CDC
	Partial	Survival and resolution of fever but persistence of at least one sign or symptom attributable to CDC: digestive signs, hepatomegaly, splenomegaly
	Progression	Survival and persistence or recurrence of fever attributable to CDC
Biological response	Complete	resolution of liver enzymes: ASAT/ALAT + alkaline phosphatase + Gamma glutamyl transferase ≤ 2N + resolution of inflammatory syndrome (C-Reactive Protein ≤ 20 mg/L)
	Partial	persistence of at least one abnormal parameter among liver enzymes > 2N, without worsening attributable to CDC or persistence of CRP > 20 mg/L, without worsening attributable to CDC
	Progression	Worsening of liver enzyme abnormalities OR CRP increase attributable to CDC
Radiological response	Complete	Complete response on each organ
	partial	Complete or partial response on each organ
	stable	partial or stable response on each organ, OR complete or partial or stable response on one organ + partial or stable response on the other one
	Progression	progression or dissociation (in the same organ) on liver and/or spleen

### Secondary outcome measure

Secondary outcome measure consists in comparison between responses to PET/CT and conventional imaging at diagnosis and M3. Responses to radiography were defined according to the Response Evaluation Criteria in Solid Tumors (RECIST1.1) (27) (for more details see Supplementary Table 2).

### Statistical analysis

The original design required a total of 100 patients with CDC. Quantitative variables were described as mean ± standard deviation (SD) in the case of normal distribution or median and interquartile range [IQR 25-75] otherwise. Comparisons for continuous data were performed through Wilcoxon test and for categorical data through Fisher’s exact test.

## Results

### Population characteristics

Among 52 patients included between November 2013 and March 2017 in 23 French onco-hematology centers, 6 were secondarily excluded for alternative diagnosis and 2 for lack of available PET/CT at diagnosis. Among 44 analyzable patients, 43 had a hematological malignancy and one underwent autologous HSCT for solid cancer (Table 2). Most of the cases (27; 61.4%) were diagnosed during the induction phase. Other comorbidities are presented in Table 2. One case had had splenectomy. Corticosteroids were prescribed for 21 (47.7%) patients in the month prior to inclusion, included in the anti-tumor chemotherapy protocol for 17 patients. The mean cumulative dose of equivalent-prednisone corticosteroids was 1264 ± 843 mg. In the month preceding CDC diagnosis, an antifungal drug was prescribed for at least 5 days in 41 patients (93.2%), 16 patients receiving it as primary or secondary prophylaxis; 18 patients were still receiving an antifungal treatment at inclusion (see Supplementary Figure 1). Invasive pulmonary aspergillosis was previously diagnosed as probable in four cases and possible in three others.

**TABLE 2:** Characteristics of 44 patients with chronic disseminated candidiasis (CDC) having a positron-emission tomography computed tomography (PET/CT) at inclusion.

	Cases (*N* = 44)
Age, mean ± SD	46.9 ± 16.3
Male gender	24 (54.6)
Body mass index, kg/m^2^	22.7 ± 4.3
Comorbidities*	
Smoking	23 (52.3)
Type II diabetes	5 (11.4)
Obstructive chronic respiratory disease	2 (4.6)
Hematological malignancies	43 (97.7)
Acute myeloid leukemia/Myelodysplastic syndrome	23 (52.3)
Acute lymphoid leukemia	15 (34.1)
Lymphoma	5 (11.4)
Solid cancer before autologuous HSCT	1 (2.3)
**Treatment phase of hematological malignancies and other treatments at CDC diagnosis^&^**	
Induction	27 (61.4)
Consolidation	8 (18.2)
Allogenic HSCT	7 (15.9)
Autologous HSCT	1 (2.3)
Corticosteroids < 1 month before inclusion	21 (47.7)
Antifungal drug at least 5 days before inclusion	41 (93.2)
Growth factors < 1 month before inclusion	30 (68.2)
**Imaging**	
Time between inclusion and PET/CT, median days, IQR 25–75	1.5 (0–4)
Time between diagnostic conventional imaging and PET/CT, median days, IQR 25–75	7 (4–12)
**Clinical and biological features**	
Fever	15 (34.1)
Liver enzyme abnormality**	9 (20.9)
C-reactive protein > 20 mg/L^$^	33 (84.6)
Leukocytes, G/L, mean ± SD	9.6 ± 7.4
Neutrophils, G/L, mean ± SD	8.0 ± 6.3
Monocytes, G/L, mean ± SD	1.0 ± 1.0
Lymphocytes, G/L, mean ± SD	0.9 ± 0.7

All variables are expressed in absolute number (percentage) otherwise indicated. SD, standard deviation; IQR, interquartile range; HSCT, hematopoietic stem cell transplantation. *None of the cases presented with chronic renal insufficiency. **Alkaline phosphatase and Gamma glutamyl transferase levels both ≥ 2N. ^$^C-reactive protein level was unknown for five patients (*N* = 39). ^&^None of the cases received leukocyte transfusion or total body irradiation.

The median duration of neutropenia before CDC diagnosis was 18 [14–24] days. The median time between CDC diagnosis by imaging and the previous period of neutropenia was 34 [24–41] days. Two (4.5%) patients were still neutropenic < 500/mm^3^ at diagnosis. Liver biopsy was performed in 13 patients in median 9 [6–12] days after CDC diagnosis; 21 (48%) patients received corticosteroids in the month before or at the time of biopsy. No granuloma or yeasts were observed. CDC was considered as probable in 20 patients on one positive BDG (*n* = 13) or recent candidemia (*n* = 7), and 24 patients had possible CDC without BDG.

Lesions were detected in liver and spleen in 29 (66%) patients, liver only in 11 (25%) patients and spleen only in 4 (9%) patients. Although the study protocol was not designed to screen for localizations other than liver and spleen, involvement of lungs and kidneys was detected in 11 and 6 patients, respectively. Antifungal treatment for CDC was ongoing at inclusion in 37 patients (84.1%). The 44 patients received antifungal drugs for CDC during the week following inclusion.

### Baseline PET/CT and conventional imaging

Positron-emission tomography computed tomography (PET/CT) was performed in median 7 [4–12] days after diagnostic imaging, while the abdominal MRI (*n* = 4) or CT (*n* = 40) was performed in median 5.5 [2.3–10] days before inclusion. All the patients had PMN ≥ 500/mm^3^ at initial PET/CT [median 5,626/mm^3^ (3,240–11,270)], except two who had leucocytes less than 1,000/mm^3^. These two patients had positive PET/CT.

**FIGURE 1:**
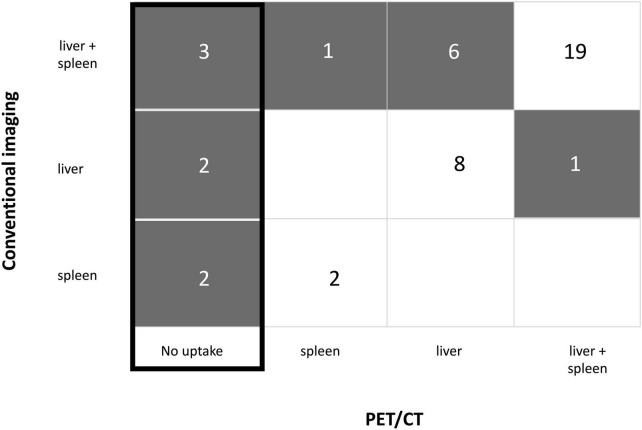
Discrepancies between baseline positron-emission tomography computed tomography (PET/CT) and conventional imaging regarding lesion localization. The number of patients is represented in each box. Gray color flags a discrepancy between PET/CT and conventional imaging.

Positron-emission tomography computed tomography (PET/CT) showed metabolic uptake in liver and/or spleen at CDC diagnosis in 37 (84.1%) patients. In addition to hepatosplenic lesions, 4 patients had metabolic uptake in lungs; uptake in kidneys was not interpretable due to renal excretion of ^18^F-FDG. Among 37 positive PET/CT, metabolic uptake matched with lesion localizations on conventional imaging in 30 (68.2%) patients (Figure 1). For 7 patients, there were discrepancies between metabolic uptake and conventional imaging. There was a lack of detection of liver and spleen lesions in one and 5 patients, respectively, but detection was better for liver lesions in one patient.

Positron-emission tomography computed tomography (PET/CT) was negative in 7 patients for whom no metabolic uptake was detected, whereas conventional imaging showed lesions on liver and/or spleen (Figure 1). There was a trend toward a longer time to implementation for the 7 negative PET/CT [15(8–17.5) days] compared to the positive PET/CT [6(4–10) days; *p* = 0.079]: 3 negative PET/CT were performed in the first week following conventional imaging and 4 PET/CT were performed at 15, 15, 20, and 23 days, respectively (see Supplementary Table 3). Among the seven patients without initial metabolic uptake, 5 had probable, and 2 had possible CDC. They had median PMN at 4,300/mm^3^ [2,510–6,425]. None had severe neutropenia. Two patients received corticosteroids in the month before inclusion and 4 patients were diabetic. Glycemia was normal at the time of PET/CT for all patients. Taking into account the fact that some data were missing in both groups, C-reactive protein (CRP) was in median 31[20–130] mg/L in the negative PET/CT group, twice lower than CRP in the positive PET/CT group [78(39–159); *p* = 0.21], three patients having CRP ≤ 20 mg/L (see Supplementary Table 3).

Regarding the number of lesions in each organ, 27 patients had > 10 metabolic lesions on liver and/or spleen and had significantly more CRP > 100 mg/L than patients with ≤ 10 lesions (respectively 54.3% vs. 20.0%; *p* = 0.049). There were no other significant differences between patients who had > 10 lesions compared to those without (Table 3). Overall, the present data do not concur to perform systematically PET/CT in the setting of suspected CDC.

**TABLE 3:** Comparison of clinical and biological parameters in patients with > 10 lesions vs. ≤ 10 lesions on baseline positron-emission tomography computed tomography (PET-CT).

	≤ 10 lesions on PET-CT (*N* = 17)	> 10 lesions on PET-CT (*N* = 27)	*P*
Age, median (IQR)	56.0 (39.3–62.5)	43.6 (31.1–58.0)	0.39
Male gender	10 (58.8)	14 (51.9)	0.76
Time between diagnostic conventional imaging and PET/CT, median days, IQR 25-75	8 (5–13)	6 (4–10.5)	0.32
HSCT	5 (29.4)	5 (18.5)	0.47
Corticosteroid use	7 (41.2)	14 (51.9)	0.55
Antifungal prophylaxis	6 (35.3)	10 (37.0)	1.0
Growth factors	9 (52.9)	21 (77.8)	0.11
Fever	4 (23.5)	11 (40.7)	0.33
Liver enzyme abnormalities*	2 (11.8)	7 (26.9)	0.28
C-reactive protein > 20 mg/L^$^	11 (73.3)	22 (91.7)	0.18
C-reactive protein > 100 mg/L^$^	3 (20.0)	13 (54.2)	**0.049**
Leukocytes > 1.0 G/L	4 (23.5)	9 (33.3)	0.74
Neutrophils > 0.5 G/L	7 (46.7)	15 (57.7)	0.53

All variables are expressed as absolute number (percentage) otherwise indicated. *Alkaline phosphatase and Gamma glutamyl transferase both ≥ 2N. ^$^C-reactive protein level was unknown for 2 patients in the group ≤ 10 lesions and 3 patients in the group > 10 lesions. IQR, interquartile range; HSCT, hematopoietic stem cell transplantation. In bold significant *P*-value < 0.05.

### M3 PET/CT and conventional imaging

**FIGURE 2:**
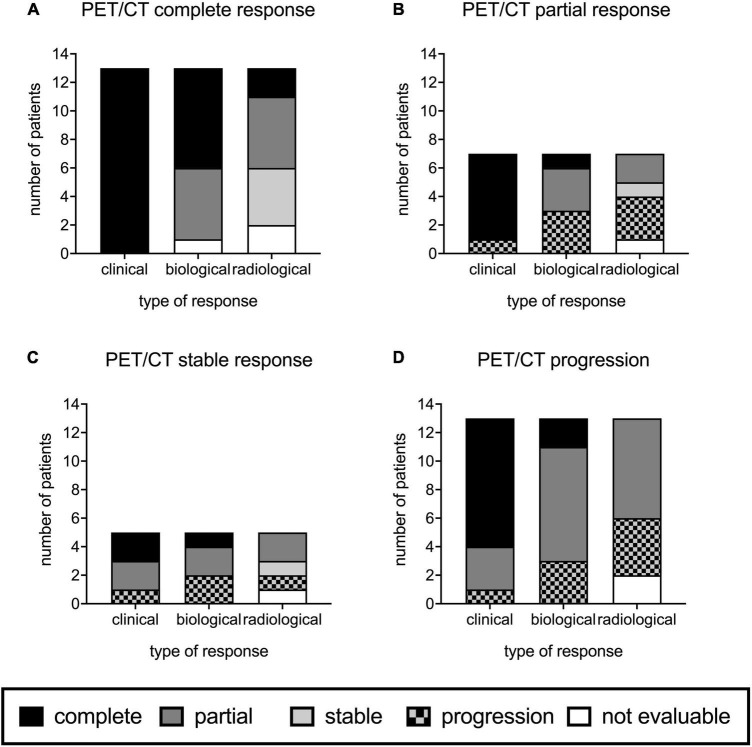
Positron-emission tomography computed tomography (PET/CT) responses at M3 compared to those of conventional imaging, clinical examination, and biology in 38 onco-hematology patients. M3 PET/CT showed complete response in 13 patients **(A)**, partial response in 7 **(B)**, stable response in 5 **(C)**, and progression in 13 patients **(D)**.

M3 PET/CT was performed in 38 patients (3 patients died before M3,3 PET/CT were missing among which 2 were negative at baseline). Figure 2 shows clinical, biological, and radiological responses according to PET/CT responses at M3.

M3 PET/CT was normalized in 13 (34.2%) patients (Figure 2A). M3 PET/CT still showed metabolic uptake at M3 in 25 (65.8%) cases: 7 had a partial response (Figure 2B), 5 had stable lesions (Figure 2C), and 13 had worsening of their lesions (Figure 2D).

M3 conventional imaging was performed in 35 patients (3 patients died before M3,6 were missing). A complete response was observed in 3 (8.6%) patients, a partial response in 18 (51.4%), stabilization of the lesions in 6 (17.1%), and worsening of abnormalities in 8 (22.9%) patients.

Among the 13 patients with normalized M3 PET/CT, 11 had still pathological conventional imaging (Figure 2A). When M3 PET/CT showed metabolic uptake (*n* = 25), no or partial improvement of the lesions on conventional imaging was noted (Figures 2B–D).

### Clinical and biological responses according to M3 PET/CT

Among the 13 patients with normalized M3 PET/CT, all had a complete clinical response and 7 had a complete biological response (Figure 2A). Corticosteroids were given for CDC treatment in 7/13 patients between inclusion and M3, and were still ongoing at M3 in 3 patients. Antifungal drugs were given less than 3 months in 5/13 patients; the median duration of treatment was 60 [50–63] days.

Among the 25 patients with persistent metabolic uptake on M3 PET/CT, a complete clinical response was observed in 17/25 (68.0%) and a complete biological response in 4 (16.0%) (Figures 2B–D). One patient had severe neutropenia < 500/mm^3^. A total of 12 patients received corticosteroids for CDC treatment and 2 received an antifungal treatment less than 3 months. PET/CT responses were not linked to hematological malignancy outcome since only one patient failed to respond to chemotherapy at M3.

### Global response at M3

Global response at M3, i.e., apyrexia and complete response to M3 PET/CT, was observed in eight patients. Among them, a complete response was also found with conventional imaging and biology in 2 and 4 cases, respectively. Of note, 5 PET/CT performed at baseline had no metabolic uptake, and stayed identical at M3. The 5 patients had a complete clinical response.

There were no significant differences between patients who had a global response at M3 and the other patients evaluable at inclusion in terms of age, gender, hematological malignancies, corticosteroids use, antifungal prophylaxis, G-CSF use, fever, liver enzyme abnormalities, CRP, white blood cell count (Supplementary Table 4).

### Evolution of radiography at M6 according to M3 PET/CT results

Among the 13 patients with normalized M3 PET/CT, 9 were evaluable for conventional imaging at M6: 5 kept an abnormal imaging and 4 had a no more lesions. Regarding the 17 patients who had persistent abnormalities on M3 PET/CT and were evaluable at M6, 12 (70.6%) still had lesions on conventional imaging.

### Outcome

Among the 44 patients, 11 (25.0%) died during the study period, 3 in the first 3 months of follow-up, 2 between M3 and M6. None of the patients with complete response to PET/CT at M3 died during the monitoring. None of the deaths were attributable to CDC: 3 were due to other infectious causes and 8 to refractory hematological malignancy.

Regarding the impact of CDC on hematological malignancy, 10 patients had allogenic HSCT during the follow-up with a median time from CDC diagnosis of 138 [102–192] days. They still received an antifungal drug at the time of HSCT in 80% of the cases. Two of them died at 38 days and 286 days, both from refractory hematological malignancy. Subsequent antitumoral chemotherapy courses were delayed in 13 patients, with a median time of 17 [8–38] days. Among the eight patients who died due to refractory hematological malignancy, only one had had a delay in chemotherapy.

## Discussion

This study is the first prospective multicentric study gathering clinical, biological, radiological and PET/CT data on a cohort of patients with CDC, now becoming as a rare disease. We did not meet our recruitment objectives, as the initial number of patients to be included in this study was 100. Changes in recommendations regarding antifungal prophylaxis in 2011 may have influenced the recruitment in our study (28). Nevertheless, data provided here allows us to get a better insight on CDC diagnosis and management.

At CDC diagnosis, discrepancies existed between conventional imaging and positive PET/CT. Indeed, PET/CT either increased sensitivity or could miss some lesions. Of note, CT was widely used in our study, although MRI is considered, at least by some authors, as the best conventional imaging to screen for hepatosplenic candidiasis lesions (29). Moreover, 16% of patients had no metabolic uptake on the initial PET/CT. PET/CT performed for infection screening can be falsely negative in the case of low inflammation due to previous prolonged anti-infectious therapy (30). In our study, 84.1% had had previous antifungal drugs before PET/CT, including the seven patients with negative baseline PET/CT, and all patients received antifungal treatment within a week following CDC diagnosis. Two other known factors that could influence ^18^F-FDG metabolic uptake are hyperglycemia and corticosteroid use. Diabetes mellitus did not seem to be associated with more negative PET/CT in the literature (31). By contrast, hyperglycemia did. Even if four patients in the negative PET/CT group were diabetic, all seven patients had normal glycemia at the time of PET/CT. Corticosteroids may attenuate ^18^F-FDG uptake in inflammatory diseases depending on treatment duration ≥ 10 days, but have a limited effect on PET/CT diagnostic accuracy (32–34). Corticosteroid were prescribed in 47.7% of our patients in the month prior to inclusion, including 2 patients with negative baseline PET/CT. Since the proportion of corticosteroid use was lower in the PET/CT negative group, corticosteroids were unlikely to have been responsible for attenuated metabolic uptake. Circulating neutrophils seemed not to influence positivity of PET/CT since all patients with negative PET/CT had full recovery of neutropenia and neutropenic patients had increased metabolic uptake. Activation of neutrophils is not always reflected by their expansion. Consequently, activated resident liver neutrophils could also play a role in increasing metabolic uptake. Low inflammatory state assessed by CRP may have led to negative PET/CT. For instance, CRP < 40 mg/L was a statistically significant predictor of negative PET/CT in suspicion of prosthetic valve endocarditis (30, 35). Our series of patients with negative PET/CT was a bit small to formally conclude whether low CRP influences metabolic uptake. However, a high CRP > 100 mg/L seemed to be associated with the presence of more lesions with metabolic uptake, and patients with negative PET/CT at baseline had lower CRP than those with positive PET/CT. PET/CT is known to have poor sensitivity for small lesions such as hepatic metastasis less than 1 cm due to a lack of resolution, which can result in false negative results (36). Criteria of inclusion in our study were based on small abscesses < 1 cm on liver and/or spleen, and were checked at inclusion. Finally, the interval between initial CDC diagnosis and PET/CT may have increased the number of negative PET/CT. There was a trend toward a longer interval between negative PET/CT and initial diagnostic imaging with a PET/CT performed in median 9 days after conventional imaging. However, a delayed PET/CT has had no impact for diagnosis of other infections such as vertebral osteomyelitis (37).

Our findings suggest that PET/CT is useful for follow-up at 3 months. Only 25% of patients with complete M3 PET/CT and clinical responses had concordant conventional imaging. In addition, liver enzyme abnormalities and inflammatory syndrome could last for months.

Dissociation between conventional imaging, clinical, and biological responses in CDC led to issues when applying criteria defining responses to therapy and study outcomes in clinical trials for IFD (25). Success was defined by complete or partial responses with radiological resolution or significant improvement or stabilization. Other studies have heterogenous combined criteria for success and failure. For instance, Kauffman et al. defined failure as persistent fever, persistence or worsening of liver lesions on CT and persisting or progressing clinical symptoms (38). Anaissie et al. added to these criteria persistence or progression of biochemical parameters (39). Finally, failure criteria in the Cornely et al. study were open to interpretation since they had been defined as unresponsive or progressing infection (40). Since responses in CDC patients could be dissociated, the response criteria chosen in this study were divided into four categories instead of success/failure, i.e., clinical, biological, radiological, PET/CT responses. New criteria to assess CDC response to treatment should be implemented to consider dissociated responses without impact on CDC prognosis.

Conventional imaging, mainly represented by abdominal CT in this study, showed long-term persistent liver and/or splenic lesions, more than 6 months for some patients, whereas clinical symptoms resolved at M3 for 75.8% of the patients. Persistent lesions on conventional imaging may encourage clinicians to continue antifungal treatment more than 3 months; as was the case for 82.9% of the patients in our study. This result is consistent with other retrospective studies with median duration of antifungal drug ranging from 96 to 210 days (2, 6, 7, 41). The recommended treatment duration in IDSA guidelines for CDC is to continue until lesions resolve on repeated imaging, which usually takes several months (4). The main reason for long-term antifungal treatment was mainly the risk of “relapse” if the treatment was withdrawn too early. Growing evidence for IRIS may explain “inflammatory rebounds” independently of fungal persistence, occurring in more than 15% of our patients. Our team has recently demonstrated that expansion of Candida-specific IFNγ-producing T cells together with features of T-cell activation and systemic inflammation supported IRIS in patients with CDC (10). Consequently, corticosteroids are more and more widely used to treat IRIS-related CDC symptoms after the initial fungal disease (6, 8, 42–44). In addition, lesions due to IRIS have shown a high metabolic uptake on PET/CT in HIV patients, possibly due to increased Glut-1 expression on T cells and monocytes (45). In this context, response to treatment in patients without symptoms of CDC at 3 months should be evaluated with PET/CT, and not CT.

Positron-emission tomography computed tomography (PET/CT) could be used to stop antifungal treatment at M3 in various IFD (46, 47). A few case reports and one retrospective study on 25 children showed the feasibility of antifungal treatment discontinuation in CDC patients using PET/CT (21, 41).

Although no death was attributable directly to CDC, chemotherapy for hematological malignancy was delayed in 20.4% of the patients. However, delayed chemotherapy could have an impact on cancer prognosis. In a recent study on 29 CDC in 639 acute myeloid leukemia patients, the long-term probability for survival was higher in CDC patients than in CDC-free patients (3). Allograft was performed despite CDC with no significant difference on overall survival between CDC and CDC-free patients. Similar conclusions arose from an older publication underlying the necessity to continue chemotherapy while treating CDC (48).

Our study had some limitations. Although it was the largest series of CDC patients, the number of patients remained small, showing scarcity of the disease. It was not surprising that half of the patients had possible CDC. CDC belonging to IRIS spectrum, mycological investigations were often performed too late compared to the initial fungal triggering event, which was most of the time undocumented during neutropenia. In our population, CDC diagnosis was performed in median more than a month after the initial neutropenic period and only 16% of the patients had initial candidemia. The now widely use of antifungal prophylaxis could have explained the longer time to diagnosis compared to ancient series (49). Even if our population had various hematological malignancies, the common point for CDC occurrence was the profound and prolonged neutropenia mainly due to intensive chemotherapy. Heterogenous time between conventional imaging and baseline PET/CT could have overrated the discrepancies.

To conclude, owing to discrepancies with conventional imaging, further studies are needed to use PET/CT for initial staging of CDC lesions. In contrast, PET/CT add clinical value in the follow-up of CDC patients. We recommend to perform a PET/CT at M3 to assess antifungal treatment response. Its usefulness for earlier, but safe, antifungal treatment discontinuation should be evaluated in further prospective studies. Whether or not PET/CT is useful before M3 is to be demonstrated.

## Data availability statement

The original contributions presented in this study are included in the article/Supplementary material, further inquiries can be directed to the corresponding author.

## Ethics statement

The studies involving human participants were reviewed and approved by CPP Ile de France 1, 2013-mai-13239. The patients/participants provided their written informed consent to participate in this study.

## Author contributions

BR and OL contributed to the conception and design of the work. CM, J-MC, SC, FM, and PrB contributed to the acquisition of the data. CE, TR, OL, and BR analyzed the data. CM, FM, SA, PiB, M-TR, TB, BR, and OL contributed to the interpretation of the data. BR, CM, CE, FM, and OL drafted the manuscript. All authors read and approved the final version of the manuscript.
