# Supplementary material for: Does 18F-FDG PET/CT add value to conventional imaging in clinical assessment of chronic disseminated candidiasis?
Source: Front Med (Lausanne). 2022 Dec 20;9:1026067. doi: 10.3389/fmed.2022.1026067 (PMC9807873; doi:10.3389/fmed.2022.1026067)

CANHPARI supplementary data

Supplementary Table 1. PET/CT criteria for CDC assessment

The intensity of ^{18}F-FDG uptake by liver and/or spleen lesions was assessed visually and measured through maximum standardized uptake value (SUV_{max}). Target hypermetabolic lesions were chosen in liver and spleen if applicable.

Complete response	Visual disappearance of all hypermetabolic target lesions (SUV_{max} < SUV_{max} relative to the background activity in the uninvolved liver). No new hypermetabolic lesion.
Partial response	Decrease ≥ 30% of SUV_{max} in hypermetabolic target lesions. No increase > 30% of SUV_{max} in target or non-target lesions.
Stable response	Neither complete, nor partial response, and absence of progression.
Progression	Increase > 30% of SUV_{max} in hypermetabolic target lesions, or increase of uptake volume, or occurrence of new hypermetabolic lesions.
Dissociation	Occurrence of new hypermetabolic lesions or increase of SUV_{max} in hypermetabolic target lesions, with disappearance or decrease of SUV_{max} in preexisting hypermetabolic target lesions.

Supplementary Table 2. Conventional imaging criteria for CDC assessment

On conventional imaging, abdominal CT or MRI, 5 target lesions were chosen per organ (liver and/or spleen) and followed on the same repeated imaging. Target lesions were defined according to the Response Evaluation Criteria in Solid Tumors (RECIST1.1). They were measurable if the longest diameter was > 10 mm, or non-measurable if the longest diameter was ≤ 10 mm.

Complete response	Visual disappearance of all target and non-target lesions.
Partial response	Decrease ≥ 30% of the number of target lesions compare to initial imaging
Stable response	Neither sufficient shrinkage for complete or partial response, and absence of progression.
Progression	occurrence of new lesions,<br>or increase ≥ 20% of number of non-measurable target lesions,<br>or increase ≥ 20% of the sum of diameters of measurable target lesions compare to baseline (initial imaging) with an absolute increase of +5 mm considering a 1-mm error per each target lesion
Dissociation	occurrence of new lesions or increase in absolute size of measurable target lesions compare to baseline (initial imaging) with an absolute increase of +5 mm considering a 1-mm error per each target lesion, in parallel with disappearance or size shrinkage of preexisting lesions.

Supplementary Table 3. Comparison of patients with and without metabolic uptake on PET/CT at inclusion

	Positive PET/CT (N=37)	Negative PET/CT (N=7)	P
**age, years**	43.6 (31.6-61.8)	58.7 (57.1-63.0)	0.10
**male gender**	19 (51.4)	5 (71.4)	0.43
**Body mass index, kg/m^2**	22.4 (19.4-24.2)	24.1 (22.6-29.2)	0.08
**Smoking**	17 (46.0)	6 (85.7)	0.10
**type II diabetes**	1 (2.7)	4 (57.1)	**0.001**
**obstructive chronic respiratory disease**	1 (2.7)	1 (14.3)	0.30
**hematological malignancies on induction/consolidation chemotherapy**	31 (83.7)	4 (57.1)	0.25
**HSCT**	5 (13.5)	3 (42.9)	
**solid cancer**	1 (2.7)	0	
**Corticosteroids <1 month before inclusion**	19 (51.4)	2 (28.6)	0.42
**antifungal drug at inclusion**	30 (81.1)	7 (100)	0.58
**Growth factors**	27 (73.0)	3 (42.9)	0.18
**Time between inclusion and PET/CT, days**	1 (-1-3)	5 (1-14)	0.06
**Time between diagnostic conventional imaging and PET/CT, days**	6 (4-10)	15 (8-18)	0.08
**Clinical and biological features at inclusion**			
**Fever**	13 (35.1)	2 (28.6)	1
**Liver enzyme abnormality*[&]**	8 (22.2)	1 (14.3)	1
**C-reactive protein, mg/L[$]**	78 (39-159)	31 (20-130)	0.21
**C-reactive protein >20 mg/L[$]**	30 (90.9)	3 (50.0)	**0.04**
**Leukocytes, G/L**	8.4 (4.6-13.5)	6.8 (3.5-7.9)	0.30
**Neutrophils, G/L**	5.9 (3.6-12)	4.3 (2.5-6.4)	0.31
**Monocytes, G/L**	0.6 (0.5-0.9)	0.5 (0.3-1.1)	0.38
**Lymphocytes, G/L**	0.7 (0.4-1.3)	0.6 (0.4-1.1)	0.80

All variables are expressed in absolute number (percentage) or median (IQR 25-75). IQR: interquartile range. HSCT: hematopoietic stem cell transplantation

* Alkaline phosphatase and Gamma glutamyl transferase both ≥2N.

[&]One patient missing in the positive PET/CT group.

[$]C-reactive protein was unknown for five patients (N=39), 4 in the positive and 1 in the negative PET/CT group.

Supplementary Table 4. Comparison of baseline characteristics of 33 evaluable patients with and without global response at M3

	Without global response N=25	With global response N=8	P
**Age, median (IQR 25-75)**	45.3 (28.0-54.2)	37.9 (31.3- 45.0)	0.58
**Male gender**	16 (64)	2 (25)	0.10
**HSCT**	5 (20)	0	0.30
**Corticosteroid use**	13 (52)	5 (62.5)	0.70
**Antifungal prophylaxis**	9 (36)	1 (12.5)	0.38
**Growth factors**	19 (76)	5 (62.5)	0.65
**Fever**	10 (40)	2 (25)	0.68
**Liver enzyme abnormalities***	6 (25)	1 (12.5)	0.65
**C-reactive protein >20 mg/L$**	21 (95)	5 (71)	0.14
**C-reactive protein >100 mg/L$**	11 (50)	2 (29)	0.41
**Leukocytes >1.0 G/L**	10 (40)	2 (25)	0.68
**Neutrophils >0.5 G/L**	16 (67)	3 (43)	0.38

All variables are expressed as absolute number (percentage) otherwise indicated.

*Alkaline phosphatase and Gamma glutamyl transferase both ≥2N.

$C-reactive protein level was unknown for 1 patient in the complete response group and 3 patients in the other group

IQR: interquartile range; HSCT: hematopoietic stem cell transplantation

Supplementary Figure 1. Antifungal drugs at inclusion in 18 patients

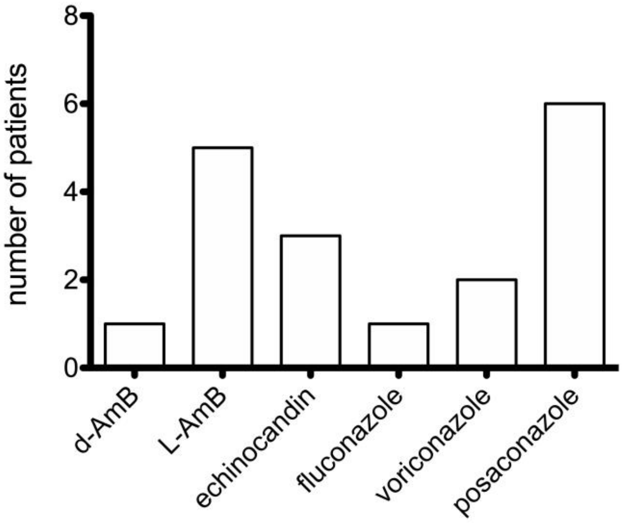

Supplement: Supplementary file 1 [file Data_Sheet_1.pdf]
